# Silicon Affects Plant Stoichiometry and Accumulation of C, N, and P in Grasslands

**DOI:** 10.3389/fpls.2020.01304

**Published:** 2020-08-27

**Authors:** Qian Hao, Shilei Yang, Zhaoliang Song, Zichuan Li, Fan Ding, Changxun Yu, Guozheng Hu, Hongyan Liu

**Affiliations:** ^1^ Institute of Surface-Earth System Science, School of Earth System Science, Tianjin University, Tianjin, China; ^2^ Key Laboratory of Recycling and Eco-treatment of Waste Biomass of Zhejiang Province, Zhejiang University of Science and Technology, Hangzhou, China; ^3^ College of Land and Environment, Shenyang Agriculture University, Shenyang, China; ^4^ Department of Biology and Environmental Science, Linnaeus University, Kalmar, Sweden; ^5^ Institute of Environment and Sustainable Development in Agriculture, Chinese Academy of Agricultural Sciences, Beijing, China; ^6^ College of Urban and Environmental Sciences, Peking University, Peking, China

**Keywords:** carbon cycles, grasslands, silicon, nutrient utilization, stoichiometry, P deficiency

## Abstract

Silicon (Si) plays an important role in improving soil nutrient availability and plant carbon (C) accumulation and may therefore impact the biogeochemical cycles of C, nitrogen (N), and phosphorus (P) in terrestrial ecosystems profoundly. However, research on this process in grassland ecosystems is scarce, despite the fact that these ecosystems are one of the most significant accumulators of biogenic Si (BSi). In this study, we collected the aboveground parts of four widespread grasses and soil profile samples in northern China and assessed the correlations between Si concentrations and stoichiometry and accumulation of C, N, and P in grasses at the landscape scale. Our results showed that Si concentrations in plants were significantly negatively correlated (*p* < 0.01) with associated C concentrations. There was no significant correlation between Si and N concentrations. It is worth noting that since the Si concentration increased, the P concentration increased from less than 0.10% to more than 0.20% and therefore C:P and N:P ratios decreased concomitantly. Besides, the soil noncrystalline Si played more important role in C, N, and P accumulation than other environmental factors (e.g., MAT, MAP, and altitude). These findings indicate that Si may facilitate grasses in adjusting the utilization of nutrients (C, N, and P) and may particularly alleviate P deficiency in grasslands. We conclude that Si positively alters the concentrations and accumulation of C, N, and P likely resulting in the variation of ecological stoichiometry in both vegetation and litter decomposition in soils. This study further suggests that the physiological function of Si is an important but overlooked factor in influencing biogeochemical cycles of C and P in grassland ecosystems.

## Introduction

Silicon (Si) is the second most abundant element in the Earth’s crust but the beneficial function of this element for plants was overlooked until studies found that Si is actively taken up by many plants (Epstein, 1994; Carey and Fulweiler, 2012; Ma and Yamaji, 2015). After being absorbed by plants dissolved Si is deposited as amorphous SiO_2_ (or phytoliths) in plant tissues (Ma and Yamaji, 2006; Cooke and Leishman, 2016), while less Si binds to semicellulose of the cell, potentially improving the strength and rigidity of plants (Broadley et al., 2012; He et al., 2015). This physiological function of Si enhances plant resistance against abiotic stresses such as drought and salt environments (Hattori et al., 2005; Liang et al., 2007; Rios et al., 2017) and biotic stresses including plant pathogens and insect pests (Ma, 2004). Si is therefore highly beneficial for plant growth and productivity (Van Bockhaven et al., 2013; Li and Delvaux, 2019).

Interest in the impacts of Si on carbon (C), nitrogen (N), and phosphorus (P) concentrations in plants is increasing (Schoelynck et al., 2010; Schaller et al., 2012a; Neu et al., 2017; Klotzbücher et al., 2018; Li et al., 2018a). Most of these studies have reported that the Si concentration is negatively correlated with that of C in plants (Schaller et al., 2012b; Klotzbücher et al., 2018). However, the application of Si promotes N concentration in non-leguminous plants exposed to N-deficient grasslands (Xu et al., 2018), despite the fact that the N concentration in wetland plants showed negative correlations with the concentration of Si (Schaller et al., 2016). Similarly, P concentration in plants in a P-deficient habitat is also promoted by the application of Si (Schaller et al., 2012b; Kostic et al., 2017; Neu et al., 2017) but is suppressed in an environment of excessive P (Ma and Takahashi, 1990; Hu et al., 2018). This shift between C concentration and nutrient status in response to Si application alters the stoichiometry of C, N, and P in plants, that this has been commonly used to indicate the balance of these elements (Hessen et al., 2004; He et al., 2008; Li et al., 2018b). Furthermore, many studies have observed that Si supply could improve plant biomass production (Eneji et al., 2008; Liang et al., 2015; Neu et al., 2017; Li et al., 2018a; Li and Delvaux, 2019; Li et al., 2019) and could enhance significantly total C, N, and P accumulation in plants (Xu et al., 2015; Li et al., 2018b). However, most of these studies were carried out in farmlands in either pot or field experiments and comprehensive investigations of natural ecosystems are still lacking.

Grassland ecosystems occupy more than 20% of the world’s land surface (Scurlock and Hall, 1998) and store considerable quantities of C to sequester 0.5 Pg C into soil every year (Scurlock and Hall, 1998; Fang et al., 2010; Wu et al., 2014). Additionally, grasslands play an important role in the global terrestrial production of biological silicon (BSi) (Blecker et al., 2006; White et al., 2012; Song et al., 2014a). However, Si is not yet well accepted as an important element in conceptual models of the grassland C biogeochemical cycle or the closely related N and P cycles (Schmidt et al., 2011; Song et al., 2012a; Lehmann and Kleber, 2015). The physiological effects of Si on accumulation and stoichiometry of C, N, and P in grasses are less well known.

We collected aboveground parts of four of the most widespread grasses in our study area in northern China to determine the concentrations of Si and explore the impacts on stoichiometry and accumulation of C, N, and P. Here, we hypothesize that Si accumulation could affect the concentration and stoichiometry of C, N, and P in grass species. The Si could promote N and P absorption in plant, while the C concentration is negatively correlated with Si concentration. We further analyzed the same species of grasses with diverse Si concentrations throughout grasslands in northern China. The study area belongs to agro-pastoral ecotone, where is experiencing degradation at varying degrees because of climate change and human activities (Jiang et al., 2006; Yang et al., 2019). These results facilitated the systematic assessment of the impacts of Si accumulation on stoichiometry and accumulation of C, N, and P in grasslands at landscape scales and further could provide references for grassland management.

## Materials and Methods

### Study Area and Field Sampling

**Figure 1:**
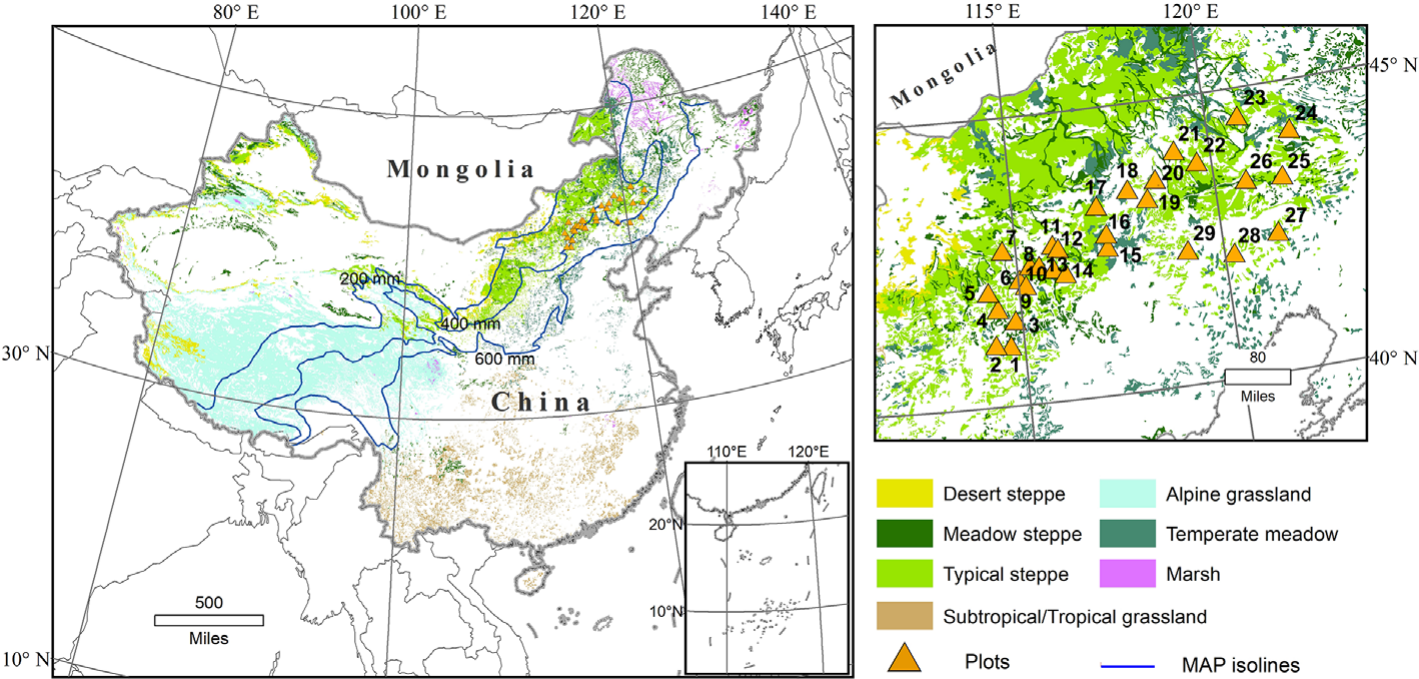
Distribution of grass types in China, with locations of the study region and sampling sites (orange triangles with numbers consistent with **Table 1**; modified from Yang et al., 2019).

The study area is situated between the northern part of Hebei Province and the southeastern part of Inner Mongolia in Northern China (**Figure 1**). The climate may be described as semi-humid and semi-arid temperate, with mean annual precipitations (MAP) and mean annual temperatures (MAT) ranging from 311.9 to 421.3 mm and from 2.1°C to 8.3°C, respectively. The main soil types are kastanozems and arenosols as classified by the Food and Agriculture Organization (FAO). The vegetation type is typical steppe or meadow steppe (**Figure 1**) (Hou, 1982).

Field investigations and sampling were conducted at 29 sites in July 2017, when grasses reached maturity. At each sampling site, three replicates of 2 m × 2 m plots were randomly set up. Data recorded for plant species included cover, abundance and height. Aboveground parts of all plants in a 1 m × 1 m quadrat within the 2 m × 2 m sample plots were harvested to estimate total aboveground biomass. At the same time, the soil profiles (0–10 cm) of each plot were collected. The detailed method could be referred to Yang et al. (2019).

Four of the most widely distributed grass species were sampled for determining aboveground biomass, including *Leymus chinensis*, *Cleistogenes squarrosa*, *Agropyron mongolicum* and *Stipa krylovii* (**Table 1**). Each species had one sample (over 150 g) in one site, but not all the sites contained all the four species (**Table 1**). A total of 57 samples were collected, including 16 samples of *L. chinensis*, 23 samples of *C. squarrosa*, 7 samples of *A. mongolicum*, and 11 samples of *S. krylovii*.

**Table 1:** Locations and environmental parameters of sampling sites with sampled plant species.

Sites	Longitude (°E)	Latitude (°N)	MAT* (°C)	MAP* (mm)	Altitude (asl, m)	Sampled species
1	114.68	41.05	3.35	405.61	1,473	*L. chinensis, C. squarrosa and*
						*A. mongolicum*
2	114.33	41.08	3.22	394.85	1,554	*L. chinensis, C. squarrosa and*
						*S. krylovii*
3	114.85	41.48	3.35	405.61	1,400	*L. chinensis, C. squarrosa and*
						*A. mongolicum*
4	114.45	41.70	3.54	344.20	1,378	*C. squarrosa and S. krylovii*
5	114.25	42.00	3.22	301.58	1,464	*L. chinensis, C. squarrosa*,
						*A. mongolicum and S. krylovii*
6	115.03	42.18	2.32	355.95	1,312	*L. chinensis and C. squarrosa*
7	114.68	42.70	3.43	282.96	1,159	*L. chinensis*
8	115.32	42.40	2.32	355.95	1,265	*L. chinensis*
9	115.17	42.07	2.32	355.95	1,452	*L. chinensis, C. squarrosa and*
						*S. krylovii*
10	115.53	42.38	2.32	355.95	1,378	*L. chinensis, C. squarrosa and*
						*A. mongolicum*
11	115.90	42.72	2.12	335.45	1,304	*L. chinensis, C. squarrosa and*
						*A. mongolicum*
12	116.03	42.65	1.64	364.19	1,338	*L. chinensis, C. squarrosa*,
						*A. mongolicum and S. krylovii*
13	115.88	42.30	2.32	367.14	1,382	*C. squarrosa and S. krylovii*
14	116.15	42.20	1.87	393.47	1,385	*C. squarrosa*
15	117.23	42.57	1.21	428.77	1,545	*A. mongolicum and S. krylovii*
16	117.23	42.80	1.21	428.77	1,432	*L. chinensis and S. krylovii*
17	117.07	43.30	1.25	404.35	1,269	*S. krylovii*
18	117.87	43.52	1.92	392.15	941	*C. squarrosa*
19	118.33	43.32	4.63	384.52	792	*L. chinensis and C. squarrosa*
20	118.58	43.63	4.87	377.80	907	*L. chinensis*
21	119.13	44.08	4.96	378.20	707	*C. squarrosa*
22	119.65	43.83	6.66	359.43	441	*C. squarrosa*
23	120.80	44.52	5.92	380.02	297	*C. squarrosa*
24	122.03	44.15	6.70	344.05	185	*L. chinensis and C. squarrosa*
25	121.67	43.37	7.17	367.59	223	*C. squarrosa*
26	120.78	43.40	7.25	352.98	295	*C. squarrosa*
27	121.35	42.42	7.80	455.02	325	*C. squarrosa*
28	120.23	42.17	6.26	430.14	650	*L. chinensis, C. squarrosa and*
						*S. krylovii*
29	119.13	42.33	7.94	386.46	573	*C. squarrosa and S. krylovii*

*MAT and MAP are obtained from the China Meteorological Data Service Center (http://data.cma.cn).

### Sample Analysis

Plant samples were carefully cleaned with distilled water, dried for 2 hours at 105°C, dried at 65°C to constant mass and subsequently finely powdered. To measure Si and P concentrations, approximately 75 mg of plant samples were fused with Li-metaborate at 950°C and dissolved in dilute nitric acid. Si and P concentrations were determined colorimetrically by the molybdenum blue method (Song et al., 2012a; Li et al., 2013). C and N concentrations (approximately 5-mg sample) were analyzed with the Elementar vario EL III (Elementar Analyzer systeme GmbH, Hanau, Germany) and each analyzed sample had three replicates.

The soil noncrystalline Si were classified into four fractions operationally based on an improved stepwise chemical extraction method (Kurtz et al., 2002; Detmann et al., 2012; Song et al., 2014b).

### Data Calculations and Statistical Analysis

At each site, to estimate the aboveground biomass of the four species we used the average importance value (IV) of each species in three repetitive sample plots to represent the percentage of the total biomass. IV of each species was calculated using the following equation:

(1) IV=(RA+RC+RH)/3

where RA represents relative abundance, RC represents relative coverage, and RH represents the relative height of the corresponding species (Liu et al., 2008). Aboveground biomass (g m^−2^) of each species was calculated using the following equation:

(2) $$\text{Biomas}{\text{s}}_{i}=\text{Total}\;\text{biomass}\times \text{I}{\text{V}}_{i}$$

where i represents *L. chinensis*, *C. squarrosa*, *A. mongolicum*, or *S. krylovii*.

The total element (C, N, or P) accumulation (g m^−2^) for each species was calculated using the following equation (taking C as an example):

(3) $$\text{Total}\;{\text{C}}_{i}\;\text{uptake}=\text{C}\;\text{concentation}\;\times \;\text{Biomas}{\text{s}}_{i}$$

where i represents *L. chinensis*, *C. squarrosa*, *A. mongolicum*, or *S. krylovii*.

One-way analysis of variance (ANOVA) was calculated to determine whether the average values of C, N, P and Si concentrations differed significantly among different species [*p* < 0.05 with the Least Significant Difference (LSD) test]. Linear regression analysis was applied to analyze the correlation (examined with Pearson’s correlation coefficients) between Si and C, N, or P for each species and all samples. In order to distinguish the independent effect of four explanatory factors (MAT, MAP, altitude, and soil noncrystalline Si) on C, N, and P accumulation in plants, the hierarchical variation partitioning (HP) was calculated for the four kinds of species and all samples in R 3.6.1 (Hao et al., 2020).

## Results

### Si, C, N, and P Concentrations and Associated Ratios in Grass Species

Si concentrations ranged from 0.23% to 2.27% among the four species (**Table 2**). Average concentrations of Si in *L. chinensis*, *C. squarrosa*, *A. mongolicum*, and *S. krylovii* were 1.12%, 1.28%, 0.83%, and 0.96%, respectively, but were not significant (*P* > 0.05) (**Table 2**). In addition, the four grasses had similar concentrations of either C, N, or P (**Table 2**). C concentrations ranged from 41.71% to 45.49% and N from 1.73% to 2.09%. P concentrations were 0.15% ± 0.03%, 0.16% ± 0.05%, 0.15% ± 0.05%, and 0.12% ± 0.04% for the four grass species. The *C. squarrosa* had significantly higher Si and P concentrations, while *S. krylovii* had significantly higher C concentration compared with other species (**Table 2**).

**Table 2:** Aboveground biomass, concentrations of Si, C, N, and P and stoichiometric ratios for the four grass species (standard deviations are given in parentheses).

Sample species	Biomass (g m^–2^)	Si (%)	C (%)	N (%)	P (%)	C:N	N:P	C:P
*Leymus chinensis* (n = 16)	7.65 (4.72)	1.12 (0.44)^ac^	43.68 (1.13)^a^	2.09 (0.41)^a^	0.15 (0.03)^ab^	21.04 (4.93)	14.84 (2.71)	327.05 (116.93)
*Cleistogenes squarrosa* (n = 23)	8.92 (6.78)	1.28 (0.39)^c^	43.38 (0.58)^a^	2.01 (0.42)^a^	0.16 (0.05)^b^	22.68 (5.96)	13.84 (5.26)	289.19 (95.51)
*Agropyron mongolicum* (n = 7)	4.60 (1.17)	0.83 (0.41)^ab^	43.77 (0.58)^ab^	1.85 (0.35)^a^	0.15 (0.05)^ab^	24.76 (5.62)	13.10 (3.19)	322.81 (91.25)
*Stipa krylovii* (n = 11)	14.58 (14.80)	0.96 (0.31)^ab^	44.37 (0.36)^b^	1.73 (0.56)^a^	0.12 (0.04)^a^	28.20 (8.81)	14.94 (2.49)	404.12 (141.20)
Total (n = 57)	8.44 (5.84)	1.12 (0.42)	43.70 (0.83)	1.96 (0.46)	0.15 (0.05)	23.54 (6.93)	14.24 (4.50)	326.12 (118.81)

Different letters of the superscripts represent the statistical significances among different species (*p* < 0.05).

C:N, N:P, and C:P ratios stoichiometry for *L. chinensis*, *C. squarrosa*, *A. mongolicum* and *S. krylovii* are shown in **Table 2**. The C:N and N:P ratios ranged from 13.62 to 48.30 and 5.22 to 24.45, respectively (**Table 2**). C:P ratios ranged from 157.34 to 699.58 with an average value of 326.12 ± 118.81.

### Relationships Between Si and Other Parameters

**Figure 2:**
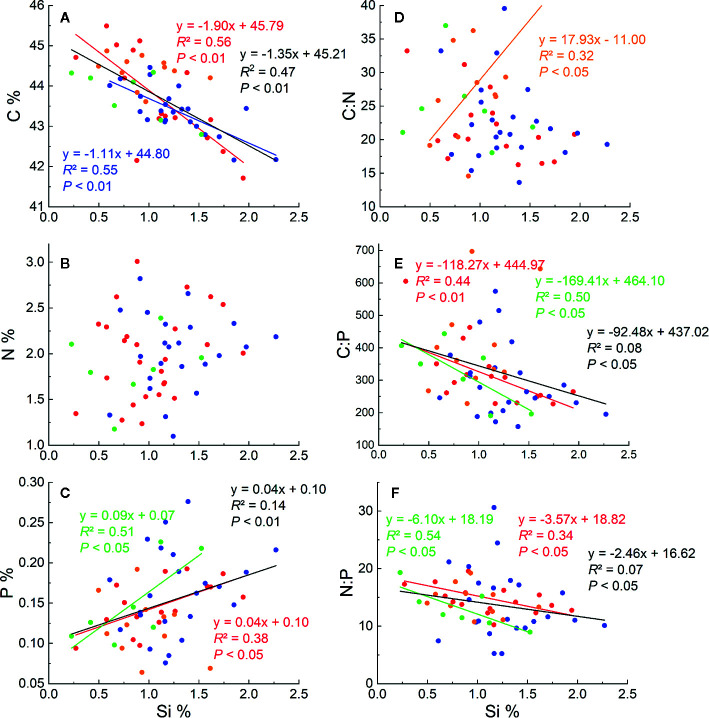
Relationships between Si concentrations and C, N, and P concentrations **(A–C)**, Si concentrations and stoichiometric ratios of N:P, C:P, and C:N **(D–F)** for these four species. The red dots, lines and text represent *Leymus chinensis*, blue represent *Cleistogenes squarrosa*, the green represent *Agropyron mongolicum* and yellow represent *Stipa krylovii.* The black dots, lines, and text represent all grass samples.

Significant negative correlations were found between Si and C concentrations in *L. chinensis*, *C. squarrosa* and for all the samples (**Figure 2A**). In contrast, Si concentrations were positively correlated with P in *L. chinensis*, *A. mongolicum*, and all the samples (**Figure 2C**). However, Si concentrations did not correlate with N concentrations both within and between all species (**Figure 2B**). Si concentration was negatively correlated with both C:P and N:P ratios for *L. chinensis*, *A. mongolicum*, and all the samples (*P* < 0.05) (**Figures 2E, F** **)**, and Si concentration was only positively correlated with C/N for *S. krylovii* (**Figure 2D**). However, a positive relationship between Si % and C:N occurred only in *S. krylovii*.

**Figure 3:**
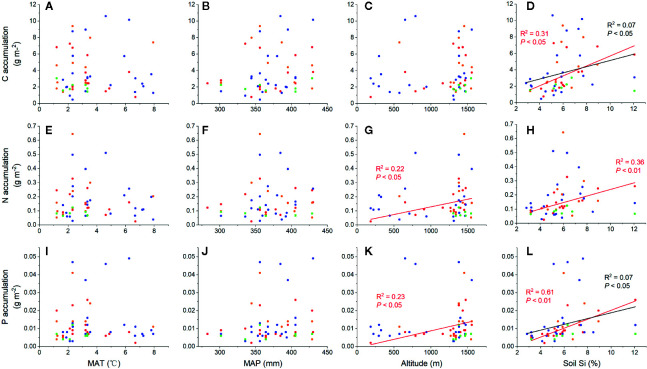
Relationships between environmental factors (MAT, MAP, altitude, and soil noncrystalline Si) and C, N, and P accumulation. The red dots, lines, and text represent *L. chinensis*, blue represents *C. squarrosa*, green represents *A. mongolicum*, and yellow represents *S. krylovii.* The black dots, lines, and text represent all grass samples. The effects of environmental factors to C accumulation **(A–D)**, N accumulation **(E–H)** and P accumulation **(I–L)**.

**Figure 4:**
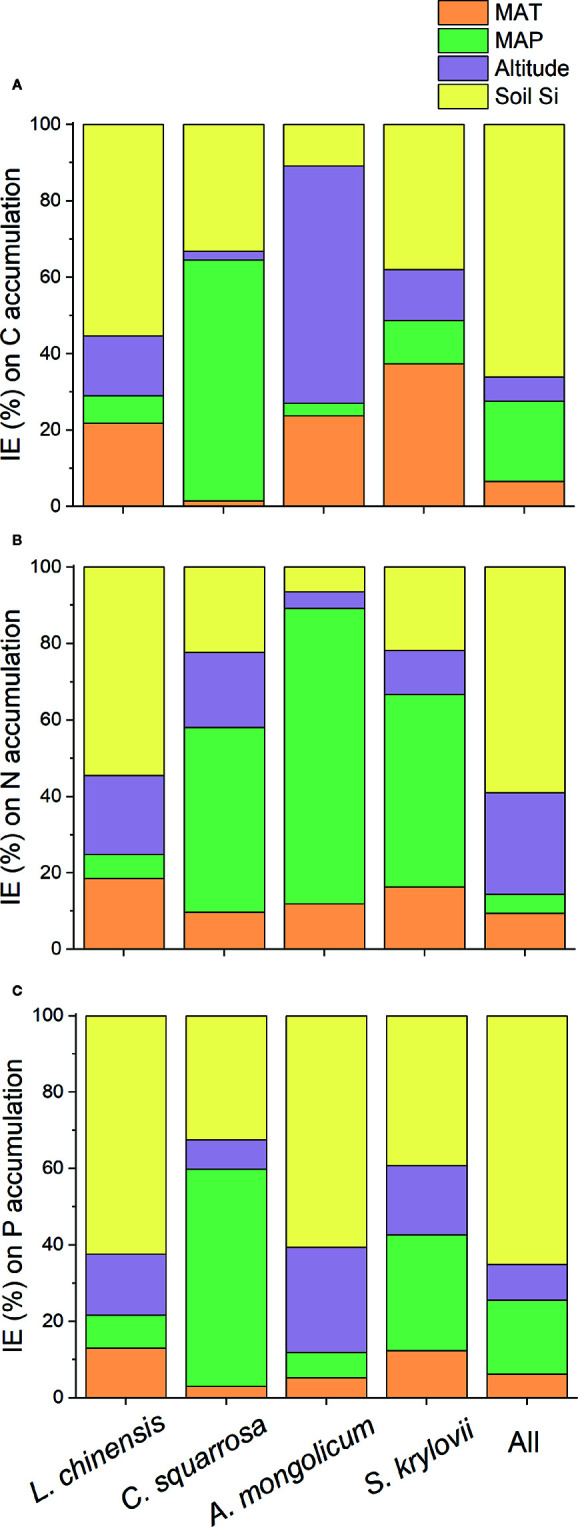
The independent effect (IE; %) of environmental factors (MAT, MAP, altitude, and soil noncrystalline Si) on C **(A)**, N **(B)**, and P **(C)** accumulation based on hierarchical variation partitioning (HP).

**Figure 5:**
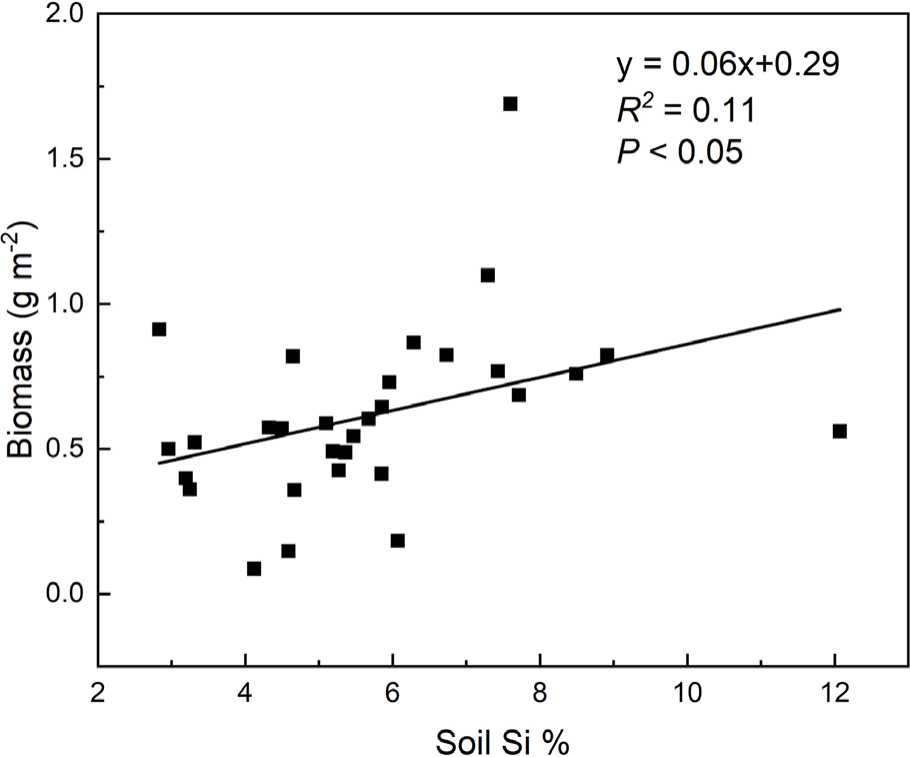
Relationships between soil noncrystalline Si concentrations and biomass of sample plots.

Strong positive correlations were observed between total C, N, and P accumulations and soil noncrystalline Si (**Figure 3**), especially for *L. chinensis* and all samples. The altitude also played an important role in N and P accumulation for *L. chinensis* (**Figures 3G, K** **)**. Based on the HP analysis, the soil noncrystalline Si had relatively higher independent effect values on the C, N, and P accumulations for *L. chinensis* and all the samples compared with MAT, MAP, and altitude (**Figure 4**). Besides, soil noncrystalline Si concentration was positively correlated with aboveground biomass (**Figure 5**).

## Discussion

### Impacts of Si on C, N, and P Concentrations in Grasses

**Figure 6:**
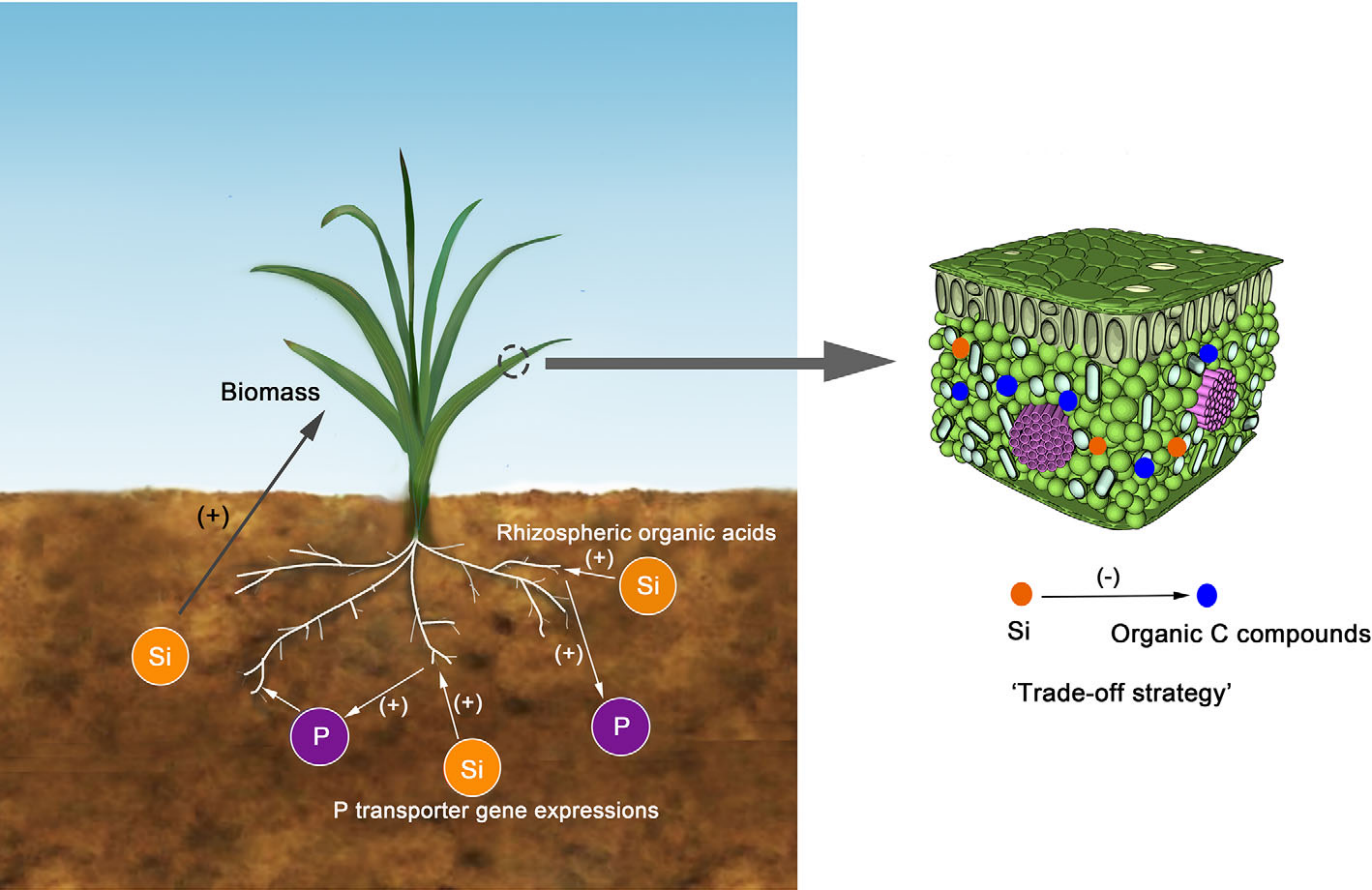
The conceptual model for the relationships between Si and C or P. The Si could substitute come organic carbon compounds, such as lignin. The Si could enhance P bioavailability in soils through regulating P transporter gene expressions or improving rhizospheric organic acids. The soil Si could enhance the biomass of plants and then positively influences the C, N, and P accumulations.

Our data revealed a significant negative correlation between Si and C concentrations in tissues of aboveground parts of the sampled grasses (**Figure 2A**). Similar results were obtained in studies of reeds (Schaller et al., 2012a; Schaller et al., 2012b), wetlands (Schaller et al., 2016), Si-fertilized winter wheat (Neu et al., 2017), and rice straw in paddies (Klotzbücher et al., 2018). Two possible reasons could explain these findings: i) the accumulation of Si may have a “diluting effect” on the concentrations of C and other elements in plants (Cooke and Leishman, 2012); ii) Si uptake in plants is a “trade-off strategy” between Si and some organic C compounds (Schoelynck et al., 2010; Klotzbücher et al., 2018). In this study, the “diluting effect” is less likely to occur due to a positive correlation between Si and P concentrations (**Figure 2C**). The Si deposition in tissue works similarly to lignin, causing plants to be resistant to environmental stresses (Epstein, 1994) (**Figure 6**). Moreover, compared to the synthesis of structural organic compounds, Si deposition is a “low energy cost” strategy for most plants (Raven, 2010; Schoelynck et al., 2010; Schaller et al., 2012a; Klotzbücher et al., 2018).

In this study, Si concentration in grass was not correlated with N concentration and this is consistent with the result of rice (Klotzbücher et al., 2018; Li et al., 2018b). This effect may be caused by the offset between the “diluting effect” and the promoting influence that cannot be quantified in this study. However, the Si concentration was positively correlated with P. Similar trends have been reported in other types of grasses (Eneji et al., 2008), reeds (Schaller et al., 2012b), and wheat (Kostic et al., 2017; Neu et al., 2017). This may be related to the interaction between Si uptake and P metabolism in plants (Kostic et al., 2017). Kostic et al. (2017) found that the application of Si could enhance P bioavailability in soils with low P concentrations through up-regulating P transporter gene expressions or improving rhizospheric organic acids (**Figure 6**).

### C:N:P Stoichiometry Regulated by Si

N and P are vital nutrients for plant growth and biosynthesis of organic matter. C:N and C:P stoichiometry can represent the plants’ nutrient status in response to varying environmental conditions (Körner, 1989; Reich and Oleksyn, 2004). Previous studies have highlighted the important roles of N and P status, climate, and phylogeny in controlling C:N:P ecological stoichiometry (Wright et al., 2004; Kerkhoff et al., 2006; He et al., 2008; Olde Venterink and Gusewell, 2010; Song et al., 2014a; Li et al., 2018b). In this study, Si concentrations in the aboveground grass tissues were negatively correlated with grass C but positively with P, resulting in a significant negative correlation between grass Si and the C:P ratio. This may indicate that Si uptake could have a profound impact on the utilization of plant nutrients (Song et al., 2014a; Li et al., 2018b).

Moreover, N:P stoichiometry can be used to assess the nutrient balance of plants and whether N or P limits plant growth at the ecosystem level (Koerselman and Meuleman, 1996; Verhoeven et al., 1996; Elser et al., 2000; Güsewell et al., 2003; Güsewell, 2004). For example, N:P < 14 indicates that N limits the growth of plants, whereas N:P > 16 indicates P limitation (Koerselman and Meuleman, 1996; Aerts and Chapin, 1999). P is considered a major growth-limiting factor in the grasslands of Northern China (Han et al., 2005; He et al., 2008). Our analysis showed that N:P stoichiometry decreased with an increasing Si concentration (**Figure 2F**), suggesting that Si may partly enable plants to govern the nutrient balance and alleviate P deficiency in the grasslands of northern China. These findings may support the promising potential role of Si in grassland management.

### Implications and Limitation of the Study

Although the biogeochemical cycles of C, N, and P influence most ecosystem processes (Chapin, 1980; Chapin et al., 1990; Hessen et al., 2004), Si plays a promising role in regulating the biogeochemical cycles of C, N, and P in grasslands (Conley, 2002; Blecker et al., 2006; Song et al., 2012a; Song et al., 2012b; Song et al., 2014a; Trinh et al., 2017). For example, the element release and CO_2_ consumption during silicate weathering and the sequestration of organic matter during the formation and accumulation phytolith in plants and soil.

In addition to these mechanisms, Si absorption from soil and deposition in grasses could also affect grassland C, N, and P cycles by controlling the synthesis of structural organic compounds and elemental stoichiometry. Increasing evidence shows that Si uptake by plants can enhance the accumulation of C and nutrient elements (Eneji et al., 2008; Song et al., 2014a; Neu et al., 2017; Li et al., 2018b). For example, Li et al. (2018a) summarized the positive effects of Si on total C accumulation in plants under different stresses in terrestrial ecosystems and found that Si-mediated recovery could potentially lead to a 35% increase in C accumulation. In line with these findings, the soil noncrystalline Si had significantly positively effect on plant C accumulation (**Figure 3**), which was caused by the significant increased aboveground biomass with total soil non-crystalline Si concentration in our study area (**Figure 5**). We suggest that the Si could promote the C, N, and P accumulation in plants, while with a certain biomass, C storage in aboveground parts of grassland plants would potentially decrease, and P storage would increase significantly due to Si uptake. However, these phenomena are not consistent among the four species in our study, and this inconsistency may result from different sample numbers and species characteristics. Besides, the insignificant relationships between climate and C, N, and P accumulation in plants might be partly caused by the relatively small differences in precipitation.

Si in soil and Si uptake in plants could not only affect C, N, and P concentrations and accumulations in fresh tissues but also influence the processes of plant litter decay since litter decomposition rates are closely related to the chemical composition of plant tissues, such as lignin concentrations and C, N, and P stoichiometry (Taylor and Parkinson, 1989; Gijsman et al., 1997; Koukoura et al., 2003). In grasslands, microorganisms in soils generally preferentially decompose plant litter with low lignin contents, low C:P ratios and high P concentrations (Zhang et al., 2008; Talbot and Treseder, 2012; Yang et al., 2014). Therefore, the positive effect of Si on the uptake of P over C as observed in the grass species in this study provides additional evidence that litter decomposition rates may increase significantly due to increased Si content (Schaller and Struyf, 2013; Marxen et al., 2016). However, the effects of increased Si incorporation on litter decay are complicated. For example, the protective effects of phytolith on litter may restrict the activity of fungal decomposers in soils (Schaller et al., 2014). Hence further research is needed to examine the relative importance of these opposing effects. Though the possible mechanism for Si regulating the C, N, or P cycles are discussed above, these are mainly based on previous researches of pot or field experiments and more mechanisms on natural ecosystems should be further investigated.

## Conclusions

In this study, we assessed the possible impacts of Si on stoichiometry and accumulation of C, N, and P in grasses over large landscape scales in northern China. Results showed that C concentrations in aboveground grass tissues (ranging from 41.71% to 45.49%) were significantly negatively correlated with Si concentrations (0.23% to 2.27%). P concentrations ranged from 0.10% to 0.20%, positively correlating with Si concentrations, while the correlation between Si and N was not significant. Additionally, the C:P and N:P ratios were significantly negatively correlated with Si concentrations. Compared with other environmental factors, soil noncrystalline Si had significant influences on plant C, N, and P accumulations. These results indicate that Si contents in soil and deposition in grasses may influence organic C synthesis and adjust nutrient utilization. We also suggest that Si deposition may promote P absorption and mitigate the limitation of P, considering that the grassland soil in northern China is generally limited in P. Si may also play a promising role in affecting C, N, and P biogeochemical cycles in the grasslands and other terrestrial ecosystems that are dominated by Si-accumulating plants.

## Data Availability Statement

All datasets generated for this study are included in the article/supplementary material.

## Author Contributions

QH and SY analyzed the data, drew the figures, and wrote the draft of the manuscript. QH and ZS designed the experiment. ZS, ZL, FD, CY, GH, and HL supplied substantial, direct, and intellectual suggestions to the manuscript. All authors contributed to the article and approved the submitted version.

## Funding

We acknowledge the support from the National Natural Science Foundation of China (41701049, 41930862 and 41571130042) and the State’s key Project of Research and Development Plan of China (2016YFA0601002 and 2017YFC0212703).

## Conflict of Interest

The authors declare that the research was conducted in the absence of any commercial or financial relationships that could be construed as a potential conflict of interest.
